# Growth Performance, Digestive Enzymes, and TOR Signaling Pathway of *Litopenaeus vannamei* Are Not Significantly Affected by Dietary Protein Hydrolysates in Practical Conditions

**DOI:** 10.3389/fphys.2018.00998

**Published:** 2018-08-14

**Authors:** Jianchun Shao, Wei Zhao, Xinwei Liu, Lei Wang

**Affiliations:** ^1^Key Laboratory of Experimental Marine Biology, Institute of Oceanology, Chinese Academy of Sciences, Qingdao, China; ^2^Laboratory for Marine Biology and Biotechnology, Qingdao National Laboratory for Marine Science and Technology, Qingdao, China; ^3^University of Chinese Academy of Sciences, Beijing, China; ^4^Department of Life Science and Biotechnology, Research Center of Hydrobiology, Jinan University, Guangzhou, China

**Keywords:** *Litopenaeus vannamei*, protein hydrolysates, growth, digestive enzymes, TOR signaling pathway

## Abstract

Protein hydrolysates have been widely reported as the protein source of aquatic feed. However, previous studies on protein hydrolysates focused on fish under experimental conditions. In this study, a 6-week feeding trial in a greenhouse was conducted to investigate the effects of partially replacing fishmeal by protein hydrolysates on growth performance, digestive enzymes, and TOR signaling pathway of *Litopenaeus vannamei* under practical conditions. This involved randomly selecting 72,000 shrimps (initial body weight 2.26 ± 0.02 g) and placing them in groups inside nine concrete tanks (L 5 m × W 5 m × H 1 m) (3 treatments × 3 replicates × 8000 individuals per concrete tank). Two isonitrogenous (430 g kg^-1^) and isolipidic (80 g kg^-1^) diets were prepared: fishmeal diet (FM) containing 400g kg^-1^ fishmeal, and protein hydrolysates diet (PH) in which 15% of the fishmeal was replaced by protein hydrolysates. A commercial diet (CD) was used as reference. The final weight (FW), percent weight gain (PWG), specific growth ratio (SGR), and total weight for each tank (TW) of *L. vannamei* fed with FM and PH diets were not significantly different (*P* > 0.05). However, shrimp fed with PH diet had significantly higher FW, PWG, SGR, and TW values than those fed with CD diet (*P* < 0.05). Trypsin activity was significantly lower in shrimp fed with CD diet, than in shrimp fed with FM and PH diets (*P* < 0.05). However, trypsin activity of *L. vannamei* fed with FM and PH diets were not significantly different (*P* > 0.05). The mRNA expression of *tor, s6k*, and *4e-bp* genes were not significantly affected between FM and PH diets, while *tor* and *s6k* expression levels of CD diet were significantly down-regulated. Based on the above data, 15% replacement of fishmeal with protein hydrolysates did not make any difference on shrimps compared with FM group. Therefore, protein hydrolysates can partially replace fishmeal as the protein source of shrimp formula feed in practical conditions.

## Introduction

The white shrimp, *Litopenaeus vannamei*, is a commercially important species and widely cultured in farms in the western hemisphere. It has become the most extensively cultured crustacean species in China due to its high nutritional value, short life cycle, and high survival rate and yield, since its introduction in 1988 (Zhao et al., 2018). The combination of high nutritional value and increasing market demands have stimulated the development of an industry focused on the production of shrimp-formulated feed (Zhao et al., 2007). Dietary proteins are primary factors influencing shrimp growth and feed costs.

As a main source of protein for aquatic formula feed, fishmeal provides high-quality animal protein, essential fatty acids, minerals, and vitamins for most high-value aquaculture species (Samocha, 2004). However, with rapid developments in aquaculture industry, the use of fishmeal as a major protein source in formulated feed has increased the demand and prices for this product. Additionally, excessive use of fishmeal in aquatic formula feeds may cause environmental pollution due to the high content of phosphorus (Song et al., 2014). Therefore, it is necessary to find alternative sources of protein in formulated feed. Research and development of different protein sources that partially or completely replace fishmeal has become an issue that must be faced and solved in aquaculture industry. In recent years, some studies have evaluated the suitability of various feed ingredients as alternative protein sources for fishmeal. Albrektsen et al. (2006) demonstrated that there was no effect on the growth, protein digestibility, and feed intake of *Gadus morhua* when approximately 50% of fishmeal was replaced by corn gluten meal and full-fat soybean meal (2:1). The results of Yan et al. (2014) suggest that the substitution of 15% of the fishmeal by soybean meal did not show significant effects on the specific growth rate and survival rate of *Sebastes schlegeli*. The results of Sun et al. (2016) indicated that 50% of fishmeal can be replaced by fermented cottonseed meal without adverse effects on the growth and feed utilization of *L. vannamei*.

However, plant protein sources are low or even absent in some bioactive molecules and some essential amino acids, and exist anti-nutritional factors (Wang et al., 2016; Shao et al., 2017). These factors can reduce the utilization of protein (Opstvedt et al., 2003; Refstie et al., 2004), affect the palatability of the feed (Shao et al., 2017), increase the feed coefficient, and even cause intestinal enteritis in aquatic animals (Krogdahl et al., 2003; Storebakken et al., 2000; Xu et al., 2012).

Small peptides are products of proteolysis, which can be absorbed and utilized directly by the intestines (Gilbert et al., 2008). Including small peptides or hydrolyzed proteins into the diet would enhance animal growth and development (Hou et al., 2017). General protein hydrolysates, which were produced from the by-products of fish or shrimp by enzymatic treatment, have proved to be a potential protein source with well-balanced amino acid profile (Chalamaiah et al., 2012; Cai et al., 2015). Several previous studies have reported that the use of protein hydrolysates as substitute for the fishmeal could improve the growth and feed utilization of aquatic animals (Cordova-Murueta and Garcia-Carreno, 2002; Plascencia-Jatomea et al., 2002; Song et al., 2014). However, our understanding of the effect of partially replacing fishmeal with protein hydrolysates, on the growth performance and digestive enzymes of *Litopenaeus vannamei*, remains limited. Previous studies on protein hydrolysates as a substitute for the fishmeal are mostly focused on experimental conditions, and its use in a practical experiment remains unknown. In this study, we evaluate the replacement of fishmeal with protein hydrolysates under practical conditions. A few studies have explored the potential mechanism of protein hydrolysates as a substitute for the fishmeal on *L. vannamei*. Target of Rapamycin (TOR) signaling pathway has proved to be closely related to nutrient sensing, metabolism, and growth in multiple species (Loewith and Hall, 2011; Weisman, 2016). It regulates protein synthesis through ribosomal protein S6 kinase (S6K) and the eukaryotic translation initiation factor 4e-binding protein (4E-BP) (Wullschleger et al., 2006). Therefore, the effect of partially replacing fishmeal by protein hydrolysates on the TOR signaling pathway is also studied in this research. Results were analyzed to assess the feasibility of replacing dietary fishmeal with protein hydrolysates, in order to provide theoretical support for the application of protein hydrolysates in shrimp feed.

## Materials and Methods

### Diet Preparation

Protein hydrolysates were obtained from Yier Biological Technology Co., Ltd. (Qinhuangdao, China). The by-products of fish were enzyme-hydrolyzed and spray-dried to produce protein hydrolysates. Proximate analyses and amino acid profile of protein hydrolysates were determined by independent laboratories and are presented in **Table 1**. Distribution of molecular weight of peptides of protein hydrolysates was analyzed in the Institute of Oceanology, Chinese Academy of Sciences, Qingdao, China (**Table 2**). Based on the preliminary experimental results, two isonitrogenous (430 g kg^-1^) and isolipidic (80 g kg^-1^) diets were prepared—one diet (FM) containing 400g kg^-1^ fishmeal and the other diet (PH) in which 15% of the fishmeal in FM was replaced by protein hydrolysates. In addition, a commercial diet (CD) obtained from commercial feed companies was used as an external control. Diets with different proportions of fishmeal were formulated as indicated in **Table 3**.

**Table 1:** Analyzation of nutritional components of protein hydrolysate (g/100 g dry weight).

Item	Content
Crude protein	62.29
Crude lipid	3.94
Dry matter	95.54
Calcium	0.25
Phosphorus	0.45
Arginine	3.93
Methionine	1.07
Tryptophan	0.60
Valine	3.83
Isoleucine	2.68
Leucine	4.62
Threonine	2.56
Phenylalanine	2.93
Histidine	1.19
Lysine	2.76
Tyrosine	1.26
Serine	4.41
Glutamic	8.05
Proline	3.57
Glycine	3.18
Alanine	3.32
Cysteine	1.05

**Table 2:** Distribution of molecular weight of peptides of protein hydrolysate.

Peptide molecular weight	Distribution (%)
<180 Da	24.6
180–500 Da	36.56
500–1000 Da	15.64
1000–5000 Da	23.08
>5000 Da	0.12

**Table 3:** Composition and proximate analysis of the experimental diets.

Ingredient (g/100g)	FM	PH	CD
Protein hydrolysate^a^	0	6.2	–
Fish meal^b^	40	34	–
Soybean meal^c^	28	28	–
Wheat flour^d^	20.3	19.8	–
Fish oil	3.7	4	–
Squid meal^e^	3	3	–
Soy lecithin	1	1	–
Brewer’s yeast	2	2	–
Vitamins premix^f^	1	1	–
Minerals premix^g^	1	1	–
*Proximate analysis*			
Moisture	9.26	9.37	9.15
Crude protein	43.31	42.87	42.19
Crude lipid	8.16	8.24	7.29
Ash	10.97	11.23	11.59
Gross energy (kJ g^-1^)^h^	17.06	16.99	16.72

*^*a*^Protein hydrolysate: 62.29% crude protein, 3.94% crude lipid, Yier Biological Technology Corporation, Qinhuangdao, China. ^*b*^Fish meal: 64.1% crude protein, 9.5% crude lipid, Dale Feed Corporation, Yantai, China. ^*c*^Soybean meal: 42.8% crude protein, 1.2% crude lipid, Dale Feed Corporation, Yantai, China. ^*d*^Wheat flour: 15.0% crude protein, 0.8% crude lipid, Dale Feed Corporation, Yantai, China. ^*e*^Squid meal: 82.8% crude protein, 4.3% crude lipid, Dale Feed Corporation, Yantai, China. ^*f*^Vitamins premix (kg^-*1*^ of diet): vitamin A, 250,000 IU; riboflavin, 750 mg; pyridoxine-HCl, 400 mg; cyanocobalamin, 1 mg; thiamin, 250 mg; menadione, 250 mg; folic acid, 125 mg; biotin, 10 mg; a-tocopherol, 2.5 g; myo-inositol, 8000 mg; calcium pantothenate, 1250 mg; nicotinic acid, 2000 mg; choline chloride, 8000 mg; vitamin D3, 45,000 IU; vitamin C, 7000 mg. ^*g*^Minerals premix (kg^-*1*^ of diet): ZnSO_*4*_ ⋅7H_*2*_O, 0.04 g; CaCO_*3*_, 37.9 g; KCl, 5.3 g; KI, 0.04 g; NaCl, 2.6 g; CuSO_*4*_⋅5H_*2*_O, 0.02 g; CoSO_*4*_⋅7H_*2*_O, 0.02 g; FeSO_*4*_⋅7H_*2*_O, 0.9 g; MnSO_*4*_⋅H_*2*_O, 0.03 g; MgSO_*4*_⋅7H_*2*_O, 3.5 g; Ca(HPO_*4*_)_*2*_⋅2H_*2*_O, 9.8 g. ^*h*^Gross energy was calculated using the factors as follows: protein, 23 kJ/g; lipid, 35 kJ/g; carbohydrates, 15 kJ/g (Molina-Poveda et al., 2013).*

All ingredients were triturated to obtain a particle size of <300 μm, and thoroughly mixed and homogenized. The 1.6-mm diameter pellets were wet-extruded using a meat grinder. The pellets were then dried in a forced air oven at 40°C to approximately 10% moisture content and stored at -20°C until used. Amino acid compositions of the experimental diets are shown in **Table 4**.

**Table 4:** Essential amino acid profile (%) of experimental diets.

Amino acid	FM	PH	Requirement
			for shrimps
Arginine	2.63	2.63	1.90^a^
Histidine	1.34	1.28	0.80^b^
Isoleucine	1.82	1.82	1.00^b^
Leucine	3.01	3.01	1.70^b^
Lysine	2.92	2.79	2.10^a^
Methionine	0.95	0.91	0.90^c^
Phenylalanine	1.84	1.86	1.40^b^
Threonine	1.67	1.66	1.40^d^
Valine	2.16	2.19	1.40^e^

*^*a*^Millamena et al. (1998). ^*b*^Millamena et al. (1999). ^*c*^Richard et al. (2010). ^*d*^Millamena et al. (1997). ^*e*^Teshima et al. (2002).*

### Shrimp and Rearing Conditions

The feeding trial was performed at an aquatic farm in Qingdao city, China. The *L. vannamei* shrimp (2.26 ± 0.02 g) were acclimatized for a week prior to the experiments, at 28 ± 1°C in oxygenated seawater (30‰ salinity). For the feeding trial, healthy shrimp were randomly divided into nine concrete tanks (L 5 m × W 5 m × H 1 m) in greenhouse with 8000 shrimps per tank. Each tank was equipped with a water exchange system and uninterrupted oxygenation system. The three diets (FM, PH, and CD) were randomly allotted in triplicate to the nine tanks. During the experiment period, the shrimp were fed four times a day at 6:00, 11:00, 17:00, and 23:00 h, for 42 consecutive days, at 5% of their body weight. Water quality parameters, including water temperature, pH, salinity, and dissolved oxygen, were measured using a multiparameter analyzer (5200A-DC, YSI, United States), and ammonia-nitrogen and nitrite-nitrogen were measured using a spectrophotometer (L53, UP GENERAL, China). The conditions of water temperature (28 ± 1°C), pH (8.0 ± 0.2), salinity (30 ppt), dissolved oxygen (> 5.5 mg L^-1^), ammonia-nitrogen (<0.30 mg L^-1^), and nitrite-nitrogen (<0.10 mg L^-1^) were ensured during the experiment period.

### Sample Collection and Growth Performance Analysis

At the end of the experiment, all shrimp were starved for 12 h to enter a basic metabolic state and eliminate the dietary effect. Twenty shrimps per tank were randomly chosen, weighed, and counted to determine the weight gain, specific growth ratio, and feed efficiency. Ten shrimps from each tank were collected and aseptically sacrificed in ice-bath, and their hepatopancreas and muscle were rapidly frozen in liquid nitrogen and stored at -80°C for digestive enzymes and gene expression level analysis. Another ten shrimps were sampled and stored at -20°C for body composition analysis. All the experiments were conducted in accordance with the recommendations in the Guide for the Care and Use of Laboratory Animals of the National Institutes of Health (NIH). The study protocol and all experimental design were conducted with approval from Experimental Animal Ethics Committee of Institute of Oceanology, Chinese Academy of Sciences.

The total weight of all the shrimps per tank was measured at the end of the experiment. Percent weight gain (PWG), specific growth ratio (SGR), and feed efficiency (FE) were calculated according to the following equations:

$$\begin{array}{c} Percent weight gain(\mathrm{PWG},\%)\;=\;100\;\times \;(W_{2}\;{-W}_{1}){/W}_{1}.\\ Specific growth ratio(\mathrm{SGR},\%{\mathrm{day}}^{-}{}^{1})\;=\;100\;\times \;({\mathrm{LnW}}_{2}{- Ln W}_{1}) / T .\\ Feed efficiency(\mathrm{FE})\;=\;(W_{2}{-W}_{1})/feed consumed. \end{array}$$

where W1 and W2 are initial and final body weight of *L. vannamei* in each tank, respectively; T is the duration of the experiment (42 days).

### Chemical Analysis of Feed and Shrimp Muscle

A standard method of Association of Official Analytical Chemists (AOAC, 2012) was used for the analysis of shrimp muscle (moisture, crude protein, crude lipid, and ash) and the experimental diets. The dry matter remaining after drying samples at 105°C was burned to ash at 550°C for 4 h. Total nitrogen content was estimated according to the Dumas method (Ebeling, 1968). Crude protein content was determined in an indirect manner (nitrogen × 6.25). Crude lipid was measured after diethyl ether extraction using Soxhlet method (Buchi 36680, Switzerland). Ash was examined after combustion in a muffle furnace at 550°C for 16 h.

### Activity Quantification of Digestive Enzymes

Hepatopancreas samples were homogenized in ice-cold water in the proportion of 1:9 (w/v), then centrifuged at 1800 g for 30 min at 4°C, and the cold hepatopancreas supernatant was used for evaluation of enzymatic activity. The activities of trypsin, α-amylase, and lipase were measured using a commercial kit (Nanjing Jiancheng Bioengineering Institute, Nanjing, China).

### Gene Expression Analysis

#### Total RNA Extraction and Reverse Transcription

Total RNA was extracted from hepatopancreas and muscle using a Reagent kit (Takara, Japan) according to the manufacturer’s instructions. The quantity and integrity of RNA were measured using NanoDrop (ND-2000, Thermo Fisher, United States) and 1.0% denaturing agarose gel. cDNA was synthesized using approximately 2 μg RNA; TransScript^®^ One-Step gDNA Removal and cDNA Synthesis Kit was used according to the manufacturer’s protocol (TransGen Biotech Co., Ltd., China). The amplification was performed by thermal cycler (A300, LongGene, China) involving the following steps: 65°C for 5 min, ice bath for 2 min, 42°C for 15 min, and 85°C for 5 s.

#### Real-Time Quantitative PCR Analysis

RNA extracted from muscle was used for expression analysis of TOR signaling pathway genes *tor, s6k*, and *4e-bp*. The specific primer sequence for Real-time quantitative PCR was designed in accordance with Shao et al. (2017), and beta-actin primers for measuring the endogenous control gene (**Table 5**). Real time PCR was performed using a quantitative thermal cycler (LineGene9600, BIOER, China) and the TransStar Top Green qPCR Supermix, according to the manufacturer’s recommendations (TransGen Biotech Co., Ltd., China). Each sample was run in triplicate. PCR amplification was performed using the following cycling conditions: denaturation for 30 s at 94°C, followed by 40 cycles of 94°C for 5 s, and 30 s at 60°C. The dissociation curve analysis was performed at the end of each PCR reaction to confirm that only one PCR product was amplified and measured. Data were analyzed with Microsoft Excel, and 2^-ΔΔCt^ method was used to analyze the expression level of different genes.

**Table 5:** Primers used for real-time quantitative PCR.

Gene name	Primer sequence (5′-3′)	Product size (bp)
*tor*	F-TGCCAACGGGTGGTAGA R-GGGTGTTTGTGGACGGA	181
*S6k*	F-GCAAGAGGAAGACGCCATA R-CCGCCCTTGCCCAAAACCT	210
*4e-bp*	F-ATGTCTGCTTCGCCCGTCGCTCGCC R-GGTTCTTGGGTGGGCTCTT	226
*β-actin*	F-GCCCATCTACGAGGGATA R-GGTGGTCGTGAAGGTGTAA	121

### Statistical Analysis

All data were checked for normality and homogeneity of variance before analysis. The growth performance, activity of digestive enzymes, and genes expression levels were subjected to one-way analysis of variance (ANOVA). If any effect was significant, the difference between the means were analyzed by Duncan’s test for unplanned multiple comparison of mean (*P* < 0.05). Data are presented as mean ± SE. All the data were analyzed using SPSS 19.0 (SPSS, Chicago, IL, United States).

## Results

### Growth Performance and Body Composition

There was a significant difference in the growth performance of *L. vannamei* fed with different diets (**Table 6**). Final weight (FW), percent weight gain (PWG), specific growth ratio (SGR), and total weight for each tank (TW) of *L. vannamei* fed with FM and PH diets were not significantly different (*P* > 0.05). However, shrimp fed with PH diet had significantly higher FW, PWG, SGR, and TW values than those fed with CD diet (*P* < 0.05). Compared to FM, PH and CD did not influence the feed efficiency (FE) of shrimps (*P* > 0.05).

**Table 6:** Growth performance of shrimps fed with different experimental diets for 6 weeks.

Parameters	Diets treatments		
	CD	FM	PH
FW (g)	6.48 ± 0.24^a^	7.14 ± 0.45^ab^	8.04 ± 0.41^b^
PWG (%)	186.79 ± 10.78^a^	215.89 ± 19.74^ab^	255.45 ± 18.21^b^
SGR (%/d)	2.51 ± 0.09^a^	2.73 ± 0.14^ab^	3.01 ± 0.12^b^
FE	0.81 ± 0.04	0.85 ± 0.01	0.86 ± 0.03
TW (kg)	41.34 ± 3.42^a^	49.92 ± 3.12^ab^	52.84 ± 2.83^b^

*Values are mean of triplicate groups and presented as mean ± SE. Values in the same row with different superscripts differ significantly (*n* = 3; *P* < 0.05). FW, Final weight; PWG, Percent weight gain; SGR, Specific growth ratio; FE, Feed efficiency; TW, Total weight for each tank. CD, Commercial diet; FM, Fish meal diet; PH, Protein hydrolysate diet.*

Muscle composition of shrimps fed with different experimental diets is presented in **Table 7**. Moisture, crude lipid, and ash contents in shrimps did not have significant differences among all dietary treatments (*P* > 0.05). However, shrimp fed with FM and PH diets had significantly higher crude protein values than those fed with CD diet (*P* < 0.05).

**Table 7:** Muscle composition of shrimps fed with different experimental diets for 6 weeks.

Parameters (%)	Diets treatments		
	CD	FM	PH
Moisture	76.43 ± 0.07	76.60 ± 0.04	75.98 ± 0.36
Crude protein	21.49 ± 0.08^a^	22.09 ± 0.09^b^	22.01 ± 0.12^b^
Crude lipid	0.76 ± 0.03	0.79 ± 0.04	0.77 ± 0.03
Ash	1.48 ± 0.04	1.50 ± 0.03	1.44 ± 0.03

*Values are mean of triplicate groups and presented as mean ± SE. Values in the same row with different superscripts differ significantly (*n* = 3; *P* < 0.05). CD, Commercial diet; FM, Fish meal diet; PH, Protein hydrolysate diet.*

### Activities of Digestive Enzymes

**FIGURE 1:**
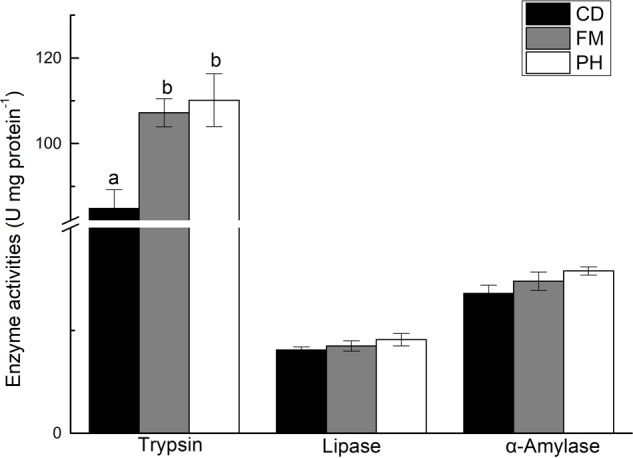
Digestive enzyme activities in hepatopancreas of shrimps fed with different experimental diets for 6 weeks. Results are shown as the mean ± SE. Different letters indicate significant differences (*n* = 3; *P* < 0.05).

There was a significant difference in trypsin activity in the hepatopancreas of *L. vannamei* fed with different diets, and no significant differences on the activity of α-amylase and lipase in shrimp fed with different diets (**Figure 1**). Trypsin activity was significantly lower in shrimp fed with CD diet, than in shrimp fed with FM and PH diets (*P* < 0.05). However, trypsin activity of *L. vannamei* fed with FM and PH diets were not significantly different (*P* > 0.05).

### Expression Levels of TOR Signaling Pathway Genes

**FIGURE 2:**
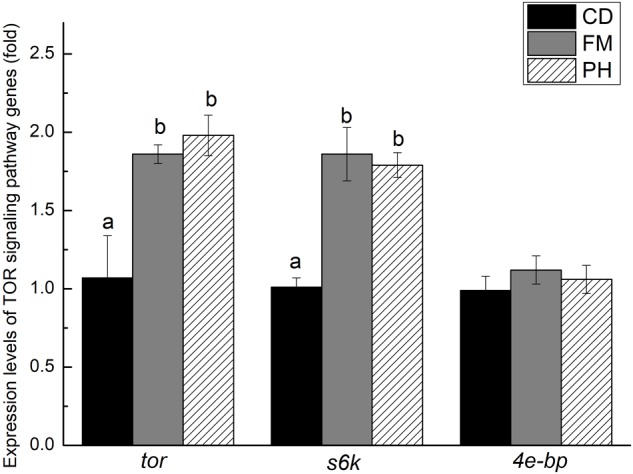
Expression levels of *tor, s6k* and *4e-bp* in muscle of shrimps fed with different diets for 6 weeks. Results are shown as the mean ± SE. Different letters indicate significant differences (*n* = 3; *P* < 0.05).

There was a significant difference in TOR signaling pathway gene expression levels in the muscle of *L. vannamei* fed with different diets (**Figure 2**). The *tor* and *s6k* expression levels of *L. vannamei* fed with FM and PH diets showed no significant difference (*P* > 0.05). However, the expression levels of *tor* and *s6k* were significantly lower in shrimp fed with CD diet, than in shrimp fed with FM and PH diets (*P* < 0.05). The expression level of *4e-bp* did not show significant differences among the three dietary treatments (*P* > 0.05).

## Discussion

In recent years, studies on the effects of protein hydrolysates on aquatic animals have been reported (Zheng et al., 2013; Khosravi et al., 2017; Quinto et al., 2018). The main sources of protein hydrolysates include two main categories: those obtained from plants and those obtained from animals. Effects of partially replacing fishmeal by plant protein hydrolysates on growth performance of aquatic animals have been reported by Song et al. (2014) and Gui et al. (2010). Song et al. (2014) demonstrated that fishmeal partially replaced with soy protein hydrolysates has no negative effect on the growth and feed efficiency of *Platichthys stellatus*. Animal protein hydrolysates as substitute for fishmeal has no negative effect on the growth performance of aquatic animals, which also has been reported by Xu et al. (2018) and Cai et al. (2015). The results of Xu et al. (2018) indicated that enzymatic hydrolysates of defatted silkworm pupa (*Bombyx mori* L.) can be included into the diet to replace 50% fishmeal of *Cyprinus carpio* var. *specularis* without negative effect on growth. Cai et al. (2015) demonstrated that ultra-filtration of fish hydrolysate replacing approximately 40% fishmeal showed no significant difference in the specific growth rate of *Larimichthys crocea.* In the present study, we got results similar to Xu et al. (2018) and Cai et al. (2015). Our results showed that no significant differences in growth performance, including FW, PWG, SGR, FE, and TW, were detected among the FM and PH groups. However, Zheng et al. (2012, 2013), and Quinto et al. (2018) reported that fish protein hydrolysates as substitute for fishmeal can improve the growth of aquatic animals. Zheng et al. (2012) reported that 20% fishmeal replaced by ultra-filtered fish hydrolysate showed the best growth, feed efficiency, and digestibility in *Paralichthys olivaceus*. Zheng et al. (2013) demonstrated that when ultra-filtered fish hydrolysate replaced 20% fishmeal, it improved the growth and feed utilization of *Scophthalmus maximus*. The difference in the effect of protein hydrolysates as substitute for fishmeal on the growth performance of aquatic animals may be related to the source and processing technology of protein hydrolysates and experimental conditions (substitution level, species, and experimental period).

The activity of digestive enzymes is directly related to the digestion and absorption of nutrients, the growth of animals, and the adaptability to the environment (Xia et al., 2018). Trypsin, lipase, and α-amylase are the main digestive enzymes in the hepatopancreas of shrimps, and they affect the digestion and absorption of food (Muhlia-Almazán et al., 2003). In the present study, we observed no significant differences in the activity of trypsin in *L. vannamei* fed with FM and PH diets, and the activity of trypsin was significantly higher than in shrimp fed with CD diet. This result was also in line with the growth performance. General proteases and trypsin from the pancreas were well related with growth (Aguila et al., 2007). Therefore, we can conclude that the difference of growth performance caused by different diets is related to trypsin activity. High trypsin activity can promote the digestion and absorption of dietary protein, thereby promoting shrimp growth. Aguila et al. (2007) reported a similar conclusion. Their results indicated that high trypsin activity can promote the digestion of food and improve the growth rate of *Octopus maya*.

The TOR pathway is a central controller of cell survival, growth, proliferation, and apoptosis (Zhao et al., 2017). The TOR signaling pathway plays an important role in balancing protein synthesis and degradation (Wullschleger et al., 2006; Sun et al., 2014). In aquatic animals, studies on the effects of nutrition level on TOR signaling pathway have been reported in fish (Seiliez et al., 2011; Li et al., 2013; Wu et al., 2017). However, the TOR signaling pathway has not been reported in *L. vannamei* fed with different diets. In this study, we investigated the expression level of TOR signaling pathway-related genes (*tor, s6k*, and *4e-bp*) in shrimp fed with different diets. In the present study, the mRNA expression of *tor, s6k*, and *4e-bp* genes was not significantly affected between FM and PH diets, while *tor* and *s6k* expression levels of CD diet were significantly down-regulated. From the results, we also found that shrimp fed with FM and PH diets had significantly higher crude protein values of muscle than those fed with CD diet. This result may be related to the regulation of protein synthesis by TOR signaling pathway. Furthermore, the variation trend of expression level of *tor* and *s6k* genes was consistent with growth performance. However, Lansard et al. (2009) reported that SGR significantly differed in *Oncorhynchus mykiss* fed with different diets, while no differences were observed in the activation of the TOR signaling pathways. Tang et al. (2013) and Zhao et al. (2012) have demonstrated that diet composition could influence TOR signaling pathway. The reason for the interesting result was not clear. Undoubtedly, more studies are needed to clarify this issue.

## Conclusion

In this study, we evaluated the replacement of fishmeal with protein hydrolysates under practical conditions for the first time. The present study showed that when 15% fishmeal was replaced by protein hydrolysates, there was no negative effect on the growth performance, digestive enzymes, and TOR signaling pathway of shrimps. Therefore, protein hydrolysates can partially replace fishmeal as the protein source of shrimp formula feed.

## Author Contributions

JS and WZ contributed to experiment design, samples collection, analysis of data, and drafting the manuscript. LW and XL also participated in designing this study and drafting the manuscript.

## Conflict of Interest Statement

The authors declare that the research was conducted in the absence of any commercial or financial relationships that could be construed as a potential conflict of interest.
